# Non-contemporaneous bilateral stem fractures occurring after staged bilateral hip revision using extensively porous-coated cylindrical femoral stems: a case report

**DOI:** 10.1186/s12891-019-2489-0

**Published:** 2019-03-18

**Authors:** Leibo Zhu, Rongxin He

**Affiliations:** 1 Department of Orthopaedics, The Second Affiliated Hospital, School of Medicine, Zhejiang University, No.88 Jiefang Road, Shangcheng Disctrict, Hangzhou, 310009 Zhejiang People’s Republic of China; 2 Department of Orthopaedics, The Second Affiliated Hospital, School of Medicine, Zhejiang University, Hangzhou, China

**Keywords:** Hip revision, Stem fracture, Porous-coated stem

## Abstract

**Background:**

Distally fixed cylindrical femoral stems extensively coated with porous materials are widely used in revision and total hip arthroplasty surgeries. Stem fracture is an uncommon complication; few case reports have been published.

**Case presentation:**

We report the case of a 51-year-old male exhibiting extensively porous-coated cylindrical femoral stems fracture after staged bilateral hip revision. His body mass index was 24.22 kg/m^2^. The major risk factor was poor proximal bony support; and femoral stems with smaller diameter.

**Conclusions:**

Certain patients are at high risk of non-contemporaneous, bilateral femoral stem fractures. It remains unclear whether fracture of the contralateral femoral stem is an inevitable fatigue fracture or reflects the increased weight imposed on the contralateral hip after the first revision. We recommend that a strut bone graft be placed to support the proximal bone, and that non-modular tapered femoral stems be employed in such patients.

## Background

Total hip arthroplasty (THA) is commonly performed to treat terminal hip joint disease. THA improves clinical outcomes and patient quality of life, and revision THA is expected to become more common in the near future. Distally fixed, extensively porous-coated cylindrical femoral stems are widely used during revision THA surgery. Here, we report the case of a 51-year-old male who underwent bilateral revision hip THA using Solution stems (DePuy, Warsaw, IN, USA) then occurred non-contemporaneous bilateral stem fractures.

## Case presentation

**Fig. 1:**
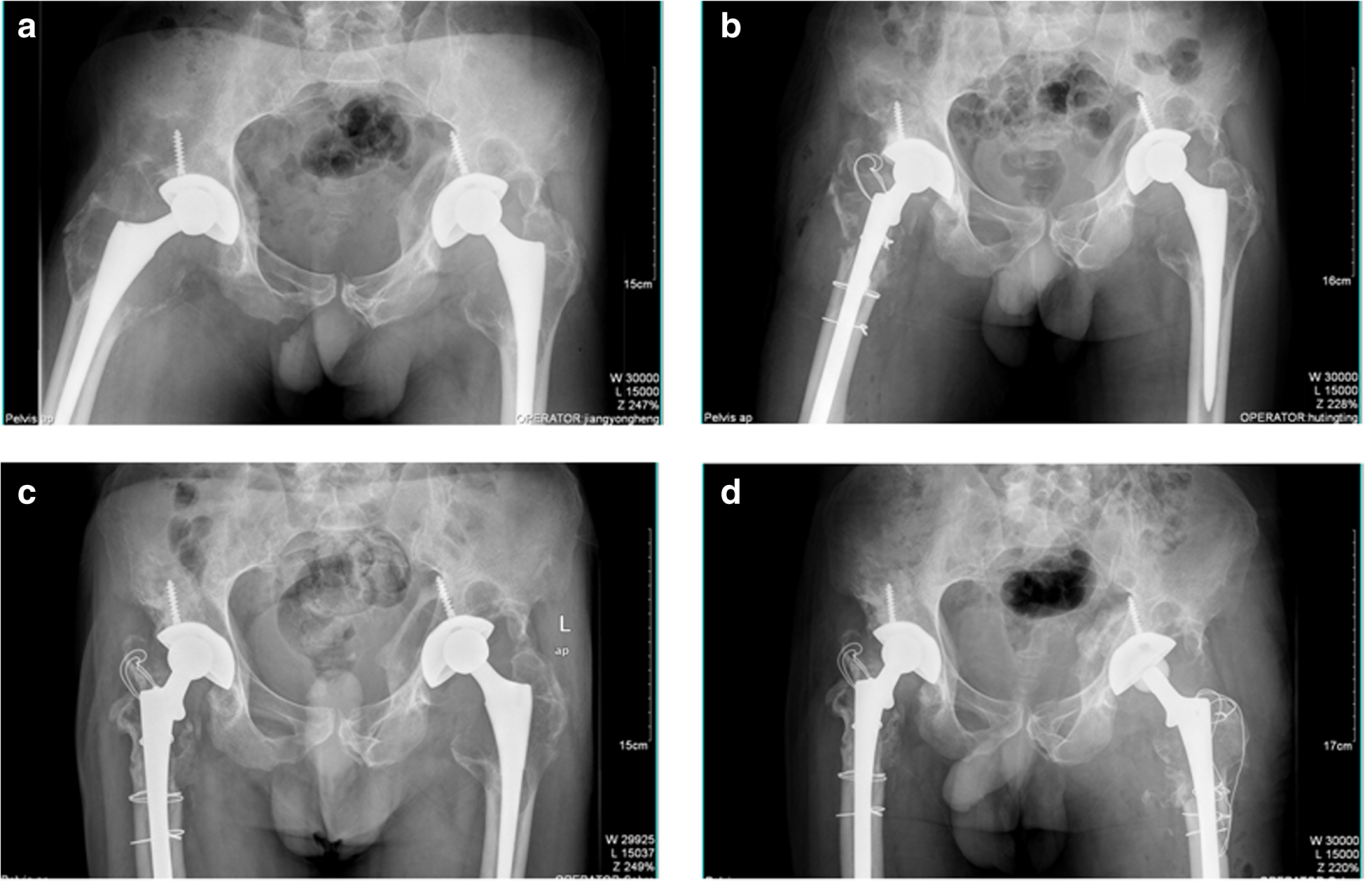
**a**: Pre-operative anteroposterior (AP) radiographs taken in June 2011. The patient had undergone bilateral total hip arthroplasty more than 10 years prior. The X-rays revealed wear of the acetabular linings of both hips, and severe osteolysis of both proximal femora and acetabula. **b**: Radiograph taken after revision of the right hip in 2011. **c**: Pre-operative AP radiograph taken in 2013. **d**: Radiograph taken after revision of the left hip in 2013

**Fig. 2:**
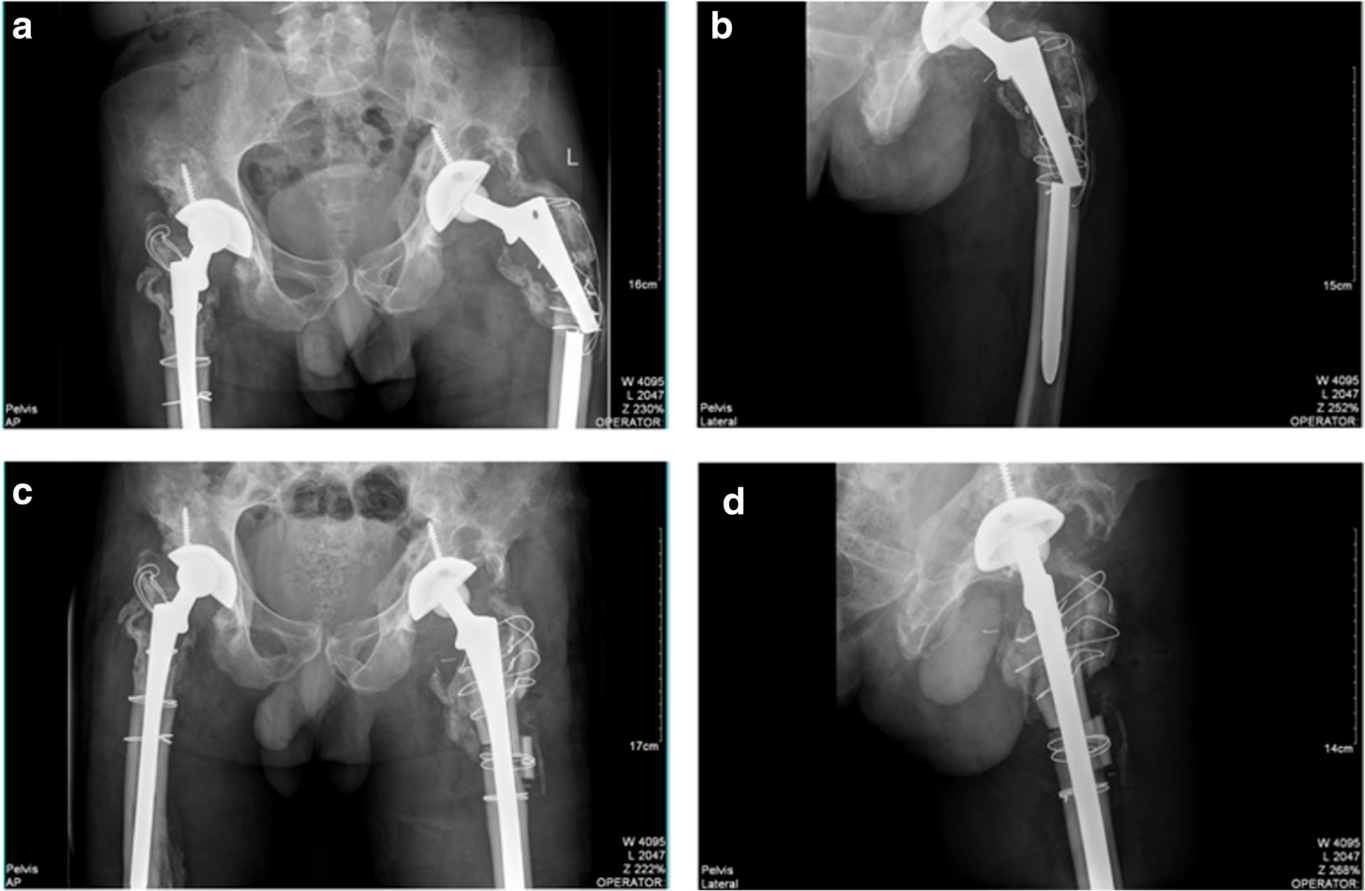
**a** and **b**: Pre-operative AP radiographs taken in December 2014 showing the left femoral stem fracture. **c** and **d**: Radiographs taken after revision of the left hip

**Fig. 3:**
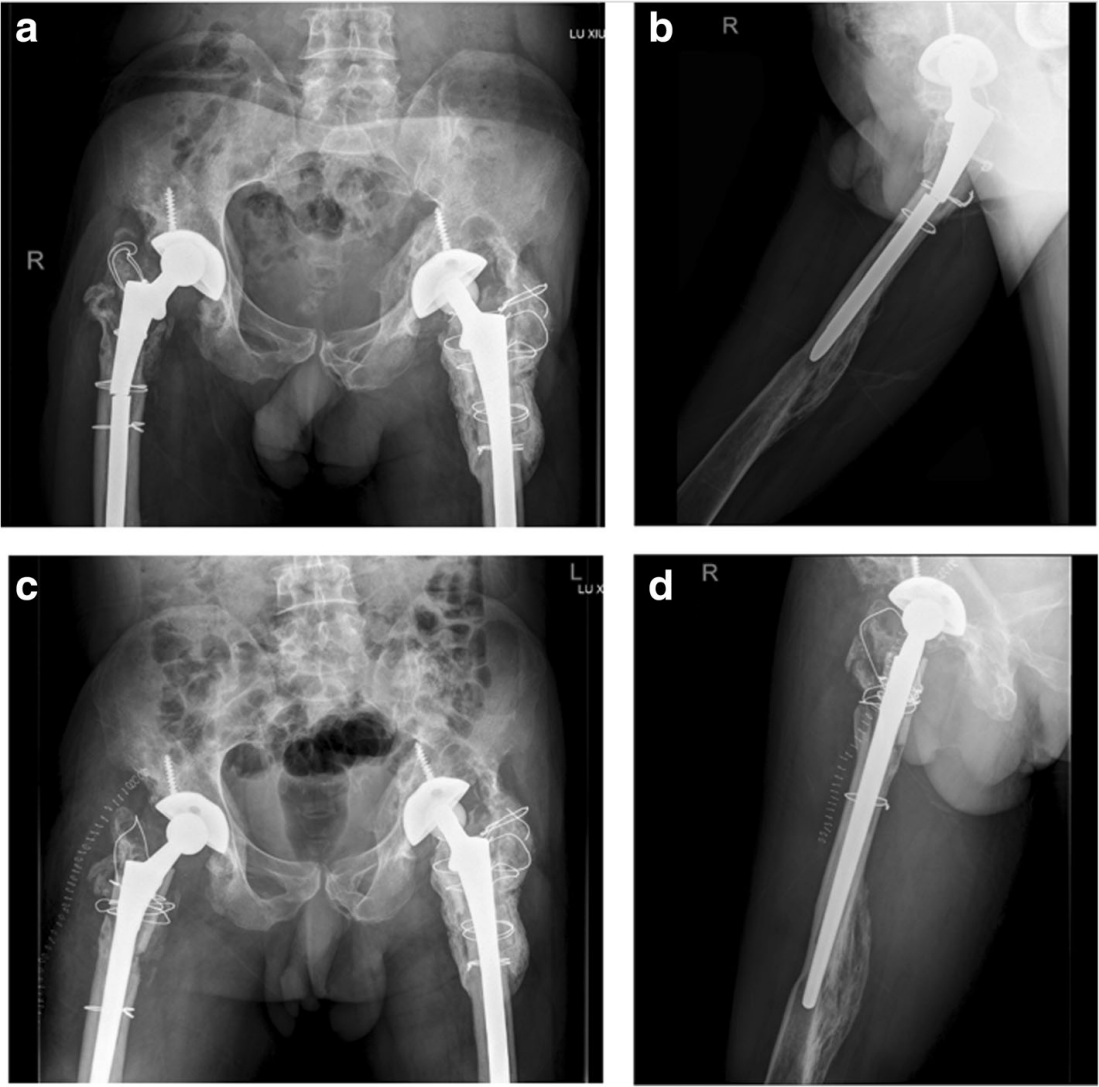
**a** and **b**: Pre-operative AP radiographs taken in September 2017 showing the right femoral stem fracture. **c** and **d**: Radiographs taken after revision of the right hip

A 51-year-old male visited us complaining of right hip pain in June 2011. He had undergone bilateral THA more than 10 years prior. X-rays revealed wear of the acetabular linings of both hips, and severe osteolysis of the proximal femora and acetabula (Fig. 1a). Preoperative laboratory data revealed no sign of infection; we thus revised the right hip on June 24, 2011. During operation, we found that the acetabular cup was firmly fixed; we thus replaced the acetabular polyethylene lining and the alumina ceramic head. The femoral stem was loose so a DePuy Solution stem (diameter 12 mm) was placed after removing the old stem. The DePuy stem is a distally fixed, extensively porous-coated cylindrical revision femoral stem (Fig. 1b). We grafted alloallergic cancellated bone onto the acetabular side; we did not graft the proximal femoral bone defect. On April 11, 2013, we revised the left hip. Again, we found that the acetabular cup was firmly fixed and the femoral stem loose. We grafted alloallergic cancellated bone onto the acetabular side, replaced the acetabular polyethylene lining and the alumina ceramic head, and again used a DePuy Solution stem (diameter 12 mm) for femoral revision (Fig. 1c and d). However, on June 122,014, the patient returned to hospital complaining of left hip pain and an inability to move after changing his sitting posture. Blood tests revealed a normal erythrocyte sedimentation rate and a C-reactive protein level of 56.8 mg/L. A stem fracture was evident on X-rays (Fig. 2a and b). We revised the left hip 11 days later; the cup and acetabular polyethylene lining were satisfactory. We replaced the femoral head and used a 14-mm-diameter DePuy Solution stem to revise the femoral side. Allograft bone augmentation of the proximal femur followed by cerclage wiring was performed (Fig. 2c and d). However, the patient returned again in September 2017 with a right femoral prosthetic fracture (Fig. 3a and b). We replaced the femoral head and the acetabular polyethylene lining, and used a 14-mm-diameter Wagner SL stem (Zimmer, Warsaw, IN, USA) to revise the right hip. We performed allograft bone augmentation of the proximal femur followed by cerclage wiring (Fig. 3c and d).

## Discussion and conclusions

Defects at the sides of the femora pose a challenge during many hip revisions. The Paprosky classification of femoral bone loss [1] suggests that when type II bone loss is encountered, placement of an extensively porous-coated cylindrical stem is appropriate. If type IIIA bone loss is evident, a stem that is < 19 mm in diameter should be extensively porous-coated cylindrical stem; if the stem is ≥19 mm in diameter, it should exhibit a modulated taper. The Paprosky classification does not mention bone grafting. Extensively porous-coated cylindrical femoral stems are reliable when used to manage femoral side defects [2]; stem fracture is an uncommon complication of hip revision. However, few such reports have appeared. Zhang et al. reported two cases of femoral stem fracture after total hip revision using extensively porous-coated cylindrical stem [3]. Busch et al. reported five stem fractures in 219 patients (2.3%) who had undergone revision THA using distally fixed, cementless extensively porous-coated cylindrical stem [4]. The risk factors included poor proximal bony support, a high body weight/body mass index (BMI), and the use of smaller-diameter (< 13.5 mm) stems.

The BMI of our patient was 24.22 kg/m^2^. We did not perform a trochanteric osteotomy when removing the prosthesis during revision. However, we placed a 12-mm-diameter stem. As the patient was young and active, insufficient bone support from the proximal femur and bony ingrowth into the distal femur may have concentrated stress on the femoral stem. Cyclic bending stresses combined with a small-diameter stem may trigger fatigue fracture. Mimura et al. reported a similar case [5], in which progressive stem bending culminated in fracture; they considered that repetitive operations/dislocations might increase the risk of such a fracture. The principal risk factors in our case were the small-diameter stem (12 mm) and inadequate proximal femoral bone support. Crowninshield et al. considered that proximal femoral bone loss, un-united fractures, and osteotomies can be expected to be most damaging to prostheses in heavier and more active patients with smaller implants [6]. Busch et al. reported reduced stress concentration around stemmed prostheses when strut bone grafts were placed on the tension sides of femora. It remains unclear whether fracture of the contralateral femoral stem is inevitable because of fatigue, or attributable simply to the increased weight borne by the contralateral hip after the first revision.

Thus, during revision hip surgery on patients exhibiting femoral bone loss, larger stems and strut bone grafts should be placed to ensure adequate proximal bone support. However, the stem diameter is obviously determined by the diameter of the femoral canal. It may not be wise to remove cortical bone to allow insertion of a larger-diameter extensively porous-coated cylindrical stem. Other options include modular/non-modular tapered stems. Modular tapered stems are thought to allow more accurate control of leg length, offset, and anteversion, thus optimising restoration of hip biomechanics. Medium-term follow-up of such patients showed good outcomes. Abdel [7] performed 519 aseptic femoral revisions using modular, fluted tapered stems; the mean follow-up duration was 4.5 years and the mean Harris hip score improved significantly. However, fractures of such stems have also been reported. Konan [8] observed five fractures at the modular junction in a series of 27 revisions. The risk factors included a high BMI, patient activity, small-diameter stems, and poor proximal bone support. Researchers try to explore the mechanism of modular stem neck fracture. Zajc [9] found that the use of an extra-long head increased the tensile stress at the neck-stem interface, triggering microcracking and, ultimately, failure of the modular stem neck.

Thus, in patients with small-diameter femoral canals and poor proximal femoral bone support, non-modular tapered stems may be the only choice; no fractures of such stems have been reported to date. The principal complications of such stems are subsidence, dislocation, and intra-operative fracture [10]. Our experience with the present case led us to conclude that the most important risk factors for femoral fractures of extensively porous-coated cylindrical femoral stem are insufficient proximal bone support and a small- diameter (< 13.5 mm) femoral stem. Strut bone grafting improves proximal bone support; non-modular, tapered femoral stems are recommended for such patients.

## Abbreviations

BMI: Body Mass Index
THA: Total hip arthroplasty
